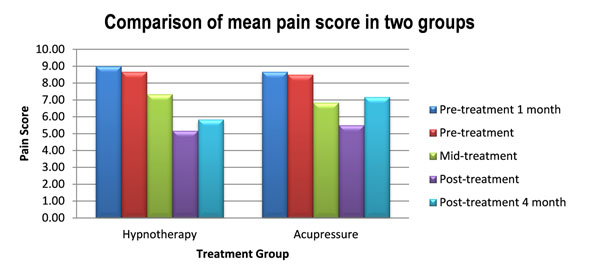# Hypnotherapy and accupressure for brachial neuralgia

**DOI:** 10.1186/1753-6561-9-S3-A82

**Published:** 2015-05-19

**Authors:** Tunku Sara Ahmad

**Affiliations:** 1 National Orthopaedic Center of Excellence for Research and Learning (NOCERAL), University Malaya, 50603, Kuala Lumpur, Malaysia

**Keywords:** hypnotherapy, acupressure, brachial neuralgia, pain management.

## Background

Pain management is concerned with the reduction of suffering and enhanced quality of life rather than a reduction of pain complaint. Persistent pain may be associated with morbidity such as emotional distress, physical disability, and sleep disturbance. Despite the prevalence of pain in musculoskeletal disorders, effective first line treatments are not without moderate to severe side effects. The aim of this study is to compare the efficacy of clinical hypnotherapy with acupressure in reducing major unresolved chronic pain; brachial neuralgia.

## Methods

A randomized controlled clinical trial in University Malaya Medical Centre (UMMC) (MEC No: 937.11) was conducted between Dec 27, 2012 and June 20, 2014. A total of 12 patients with chronic brachial neuralgia were randomly assigned to hypnotherapy group or acupressure group. Four sessions of hypnosis were conducted approximately 1 week apart in hypnotherapy group and two sessions fortnightly in acupressure group. Patients were scored during one month pre-treatment, pre-treatment (week 0), mid-treatment (week 2), post-treatment (week 4) and 4 month post-treatment. Pain scores were obtained before treatment as a baseline and post treatment as outcomes using the Visual Analogue Score (VAS), Brief Pain Inventory, and SF36v2 Health Survey which measure overall health improvement and the efficacy of the treatment were used. The hypnotherapist used a standardized pain analgesic protocol throughout the sessions and tailored induction with specific analgesia suggestions according to patient’s need. Patients in acupressure group were guided by qualified acupuncturist on how to apply the acupressure patches to the meridian points. Patients were provided with 2 weeks supply of acupressure patches and reviewed after mid treatment week 2 for any revision of meridian points patching protocol.

## Results

There was significant improvement (P=0.002) in the average pain intensity from pre-treatment mean score of 8.58 ± 1.51 (range 7-10) to post treatment 5.33 ± 2.23 (range 2-9) overall. Generally, there was 32.5% documented improvement in the pain score post-treatment as compared to pre-treatment. Hypnotherapy provided higher reduction of pain intensity of 35% (P=0.006) as compared to acupressure 30% (P=0.023) with mean score of pre treatment 8.67 ± 1.75 (range 6-10) to post treatment 5.17 ± 1.60 (range 4-8) and pre treatment 8.50 ± 1.38 (range 7-10) to post treatment 5.50 ± 2.88 (range 2-9), respectively. The comparative background variables between two groups were made on the basis of paired t-test and a non-parametric method, Wilcoxon sign rank test which was used to detect significance difference (P=0.027) and assess the efficacy of treatment between the two groups.

At the 4 month follow up assessment, the mean of pain score in hypnotherapy group (5.83 ± 1.83) was still significantly lower than that in the acupressure group (7.16 ± 2.32).

## Discussion

The majority of the patients had significant improvement in the pain intensity score except for 2 brachial neuralgia patients in the acupressure group. There was also improvement in pharmacotherapy usage (P<0.05) in terms of reduced dosage and frequency. 75% showed positive response in both treatment groups and were in favor to continue the sessions. The improvement in pain and disability score 4 months post-treatment for both groups were significantly reduced but significantly higher than the post treatment pain intensity indicating the chronic pain was successfully managed by the patient through self-hypnosis and acupressure carry over effect. The positive response to the majority of pain interference in the hypnotherapy group may be attributed to the effect of tailored suggestions accorded by suggested standardized protocol by our qualified hypnotherapists. We could not specifically determine the standard acupressure meridian point protocol for brachial neuralgia patients with neuropathic pain as the meridian points differed from patient to patient.

## Conclusions

The results suggest that both hypnotherapy and acupressure provide effective relief of average pain intensity with greater relief in the hypnotherapy group as compared to the acupressure group, in terms of pain intensity and disability score. The self-hypnosis was beneficial in term of long-term pain intensity reduction as well as quality of life. Findings in this study supported that both hypnotherapy and acupressure had the impact of improving sleep, enjoyment of life, general activity, work as well as controlling mood.

**Figure 1:**